# Traditional Risk Factors Are More Relevant than HIV-Specific Ones for Carotid Intima-Media Thickness (cIMT) in a Brazilian Cohort of HIV-Infected Patients

**DOI:** 10.1371/journal.pone.0117461

**Published:** 2015-02-18

**Authors:** Antonio G. Pacheco, Beatriz Grinsztejn, Maria de Jesus M. da Fonseca, Ronaldo I. Moreira, Valdiléa G. Veloso, Ruth K. Friedman, Marilia Santini-Oliveira, Sandra W. Cardoso, Melissa Falcão, José G. Mill, Isabela Bensenor, Paulo Lotufo, Dóra Chor

**Affiliations:** 1 FIOCRUZ, Programa de Computação Científica, Rio de Janeiro, Brazil; 2 FIOCRUZ, Instituto de Pesquisa Clínica Evandro Chagas, Rio de Janeiro, Brazil; 3 FIOCRUZ, Departamento de Epidemiologia e Métodos Quantitativos em Saúde, Rio de Janeiro, Brazil; 4 Federal University of Espírito Santo, Departamento de Ciências Fisiológicas, Vitória, Brazil; 5 University of São Paulo, School of Medicine, São Paulo, Brazil; Medical University Innsbruck, AUSTRIA

## Abstract

**Background:**

Combination antiretroviral therapy (cART) had a dramatic impact on the mortality profile in human immunodeficiency virus (HIV) infected individuals and increased their life-expectancy. Conditions associated with the aging process have been diagnosed more frequently among HIV-infected patients, particularly, cardiovascular diseases.

**Methods:**

Patients followed in the Instituto de Pesquisa Clínica Evandro Chagas (IPEC) prospective cohort in Rio de Janeiro were submitted to the general procedures from the Brazilian Longitudinal Study of Adult Health, comprising several anthropometric, laboratory and imaging data. Carotid intima-media thickness (cIMT) was measured by ultrasonography, following the Mannheim protocol. Linear regression and proportional odds models were used to compare groups and covariables in respect to cIMT. The best model was chosen with the adaptive lasso procedure.

**Results:**

A valid cIMT exam was available for 591 patients. Median cIMT was significantly larger for men than women (0.56mm vs. 0.53mm; p = 0.002; overall = 0.54mm). In univariable linear regression analysis, both traditional risk factors for cardiovascular diseases (CVD) and HIV-specific characteristics were significantly associated with cIMT values, but the best multivariable model chosen included only traditional characteristics. Hypertension presented the strongest association with higher cIMT terciles (OR = 2.51; 95%CI = 1.69–3.73), followed by current smoking (OR = 1,82; 95%CI = 1.19–2.79), family history of acute myocardial infarction or stroke (OR = 1.60; 95%CI = 1.10–2.32) and age (OR per year = 1.12; 95%CI = 1.10–1.14).

**Conclusions:**

Our results show that traditional cardiovascular disease (CVD) risk factors are the major players in determining increased cIMT among HIV infected patients in Brazil. This finding reinforces the need for thorough assessment of those risk factors in these patients to guarantee the incidence of CVD events remain under control.

## Introduction

The introduction of combination antiretroviral therapy (cART) had a dramatic impact on human immunodeficiency virus (HIV)-infected individuals mortality profile and their life-expectancy. While the pre-cART era was marked by AIDS-related conditions as leading causes of death, the current panel shows a more diverse mortality profile. Changes include an important rise in non-AIDS-related-malignancies and other end-organ diseases such as end-stage liver and renal diseases and cardiovascular diseases [1–3]. In Brazil, we have previously shown that same change in mortality profile in two large cohorts in Rio de Janeiro [4,5] and confirmed this trend in a nation-wide study dealing with associated causes of death [6].

Indeed, as life-expectancy increases, and HIV-infection becomes a chronic condition, other risk factors, such as smoking, eating habits, socio-demographic characteristics and the effects of aging start to play a more important role in this population. As a consequence, conditions associated with the aging process have been diagnosed more frequently among HIV-infected patients, including obesity, diabetes mellitus, metabolic syndrome and, particularly, cardiovascular diseases [7,8].

Several studies have shown that cardiovascular diseases in HIV-infected individuals might also be associated with the physiopathology of the infection itself and with its treatment [9–13]. Therefore, these patients require more individualized risk assessment, that goes beyond traditional ones [14–17].

Carotid intima-media thickness (cIMT) is traditionally associated with atherosclerotic lesions in the general population and has been used as a marker for early cardiovascular events [18]. In the search for an atherosclerosis marker in HIV patients, cIMT has been shown in several studies to reflect accurately atherosclerotic lesions and disease progression [13,19,20].

The association between cART treatment and the occurrence of cardiovascular events still remains controversial. It has been demonstrated that the use of contemporary antiretroviral therapy is associated with a large benefit in terms of survival that is not overcome by any increase in the rates of cardiovascular or cerebrovascular events or related mortality. In addition, suppressing HIV replication below clinical thresholds is associated with slower progression of atherosclerosis [21,22].

Even though there are already a considerable number of studies in developed countries that show the association between HIV infection itself and cIMT and several other factors associated with increased cIMT among HIV-infected patients, very few data have been published to date in low and middle income countries, including the Brazilian population [23,24], where patients present several contextual differences as compared to high income countries population such as socioeconomic and psychosocial aspects, which affect access to the health system and availability of medication as well as the prevalence of atherosclerosis traditional risk factors. In this study we describe variables associated with cIMT in HIV-infected patients in a large sample of the Instituto de Pesquisa Clínica Evandro Chagas (IPEC) cohort in Rio de Janeiro, Brazil.

## Methods

A representative sample of 649 patients followed in a prospective cohort in an HIV/AIDS referral center in Rio de Janeiro (IPEC) were included in this study. Procedures of the cohort are described elsewhere [25]. Selected patients were submitted to an extensive pre-validated questionnaire and a thorough laboratory panel. The study was approved by the Comitê de Ética em Pesquisa from IPEC, the local institutional review board. The general procedures followed those described for the Brazilian Longitudinal Study of Adult Health (ELSA-Brasil) and are described elsewhere [26]. Briefly, after signing an informed consent form, patients attending to the Investigation Center where the questionnaire was applied during an interview. A fasting venous blood sample was collected and sent to a central laboratory where all chemicals were determined. A thorough physical examination was performed, comprising anthropometry, resting blood pressure and imaging tests including carotid artery intima-media thickness measurements. Moreover, HIV cohort-specific lab tests were also obtained, including use of cART, use of PI-containing regimens, use of NNRTI-containing regimens and the time in years on each regimen, baseline CD4 counts, viral loads, cytomegalovirus (CMV) antibodies and high-sensitivity C-reactive protein (hs-CRP). Nadir CD4 counts and previous HIV viral load measurement were obtained from the cohort database, with data routinely collected from patients’ medical charts. Details of those procedures can be found elsewhere [25].

Several variables were then defined or calculated from the questionnaire and lab results. Diabetes mellitus (DM) was defined for those showing glycated hemoglobin >6.5%, fasting glucose > 126 mg/dL, oral glucose tolerance test levels > 200 mg/dL or current use of hypoglycemic drug. Resting blood pressure was measured three times in the non-dominant arm in the sitting position and hypertension was defined if the mean of the last 2 measurements showed systolic blood pressure > 140 mmHg or diastolic blood pressure > 90 mmHg or the use of anti-hypertensive drugs. Dyslipidemia was defined as low density lipoprotein (LDL) levels > = 130 mg/dL or use of lipid-lowering drugs. Cumulative smoking was calculated as pack-years, by multiplying the number of packs smoked per day by the number of years of smoking. Family history of sudden death and family history of acute myocardial infarction (AMI) or stroke were defined as the occurrence of any episode of those conditions among parents or brothers younger than 65 years of age. History of CVD was defined as past occurrence of angina, AMI or stroke. Body mass index (BMI) was calculated as the ratio of weight in kilograms and squared height in meters [27]. Cumulative viral load (VL; viremia copy-years) was defined as the area under the curve for two consecutive viral load measurements over time, in years, as described elsewhere [28]. Calculated variables were also employed and included 10-year Framingham Score Risk for general CVD [29] and creatinine clearance, calculated with the CDK-Epi formula [30] taking into account serum creatinine, age, sex and race.

The main outcome, cIMT, was measured by carotid ultrasonography of 1-cm portions of the distal left and right common carotid artery far walls with a linear transducer (nominal center transducer frequency of 7.5 MHz) (Aplio XG) with axial resolution of approximately 0.10 mm, and calculated automatically by Medical Imaging Applications software (MIA, Coralville, Iowa) over 3 cardiac cycles [26]. These procedures followed the Mannheim consensus [31]. The average of the final result for the left and right arteries was used as the outcome.

General descriptions of variables were performed in respect to calculated terciles of cIMT values and compared with Kruskal-Wallis tests for continuous and chi-squared or Fisher exact tests for categorical variables.

Linear regression models were used to study the association of all covariables with cIMT. Since cIMT values were much skewed, we chose to use a Box-Cox transformation [32] with lambda = -1.46. In order to choose the best model to explain the cIMT values we used the adaptive lasso procedure, which performs simultaneous estimation for variable selection [33]. After the best model was chosen, first order interactions were tested among variables and model fitness was inspected through residual analysis, which showed that all the models used were adequate. Because the interpretation of the betas in the Box-Cox transformed dependent variable is very difficult, we chose to also fit a proportional odds model using the same variables selected in the final linear model, using the cIMT terciles as the dependent variable, so the association strengths of the studied variables would be more easily studied and related to the descriptive statistics in Table 1.

All analyses were performed with the R environment version 3.0.3 [34].

**Table 1:** Characteristics of patients stratified by cIMT terciles ^a^.

cIMT terciles				
	0.367–0.507mm	0.507–0.589mm	0.589–1.54mm	Total	P value
Total	197	197	197	591	
Sex: Male	103 (52.28)	110 (55.84)	126 (63.96)	339 (57.36)	0.0559
Age in years—median(IQR)	36.56 (30.38,42.88)	42.89 (37.5,49.62)	50.17 (45.36,56.74)	43.79 (36.51,50.85)	< 0.001
Schooling: <9 years	91 (46.19)	94 (47.72)	98 (49.75)	283 (47.88)	0.7781
Race: black	127 (64.47)	125 (63.45)	124 (62.94)	376 (63.62)	0.9501
Smoking					< 0.001
Never	119 (60.41)	106 (53.81)	72 (36.55)	297 (50.25)	
Former	43 (21.83)	45 (22.84)	71 (36.04)	159 (26.9)	
Current	35 (17.77)	46 (23.35)	54 (27.41)	135 (22.84)	
Packs.year—median(IQR)	0 (0,4.5)	0 (0,7.62)	5 (0,20)	0 (0,11)	< 0.001
Categorical					< 0.001
<10	164 (83.25)	152 (77.55)	114 (57.87)	430 (72.88)	
10+	33 (16.75)	44 (22.45)	83 (42.13)	160 (27.12)	
Family History of Sudden Death	29 (14.72)	41 (20.81)	43 (21.83)	113 (19.12)	0.1523
Family History of AMI or Stroke	40 (20.3)	58 (29.44)	66 (33.5)	164 (27.75)	0.0112
History of CVD	8 (4.06)	14 (7.11)	24 (12.18)	46 (7.78)	0.0098
Total cholesterol (mg/dL)—median(IQR)	171 (146,203)	185 (157,210)	192 (166,221)	183 (156,211)	< 0.001
HDL (mg/dL)—median(IQR)	41 (35,49)	44 (34.75,52)	42 (35,52)	42 (35,52)	0.2424
Use of lipid-lowering drugs	16 (8.12)	30 (15.23)	35 (17.77)	81 (13.71)	0.0156
Hypertension	33 (16.75)	45 (22.84)	109 (55.33)	187 (31.64)	< 0.001
Waist circumference (cm)- median(IQR)	84 (76.3,92.4)	84.5 (77.5,94.2)	90 (83.2,97.5)	86.4 (78.2,94.5)	< 0.001
BMI—median(IQR)	24.13 (21.53,26.72)	24.34 (21.56,27.2)	25.12 (22.84,28.27)	24.39 (21.91,27.3)	0.0054
DM	44 (22.34)	45 (22.84)	66 (33.5)	155 (26.23)	0.0174
LDL (mg/dL)—median(IQR)	100.5 (85,125.45)	108 (89,131.75)	112 (91,140)	107 (87,134.5)	0.0025
Dyslipidemia	53 (27.6)	74 (38.54)	95 (48.97)	222 (38.41)	< 0.001
hs-CRP (mg/dL)—median(IQR)	0.26 (0.18,0.59)	0.3 (0.17,0.64)	0.31 (0.17,0.68)	0.28 (0.17,0.63)	0.6973
Trigicerides (mg/dL)—median(IQR)	109 (76,157.25)	120 (84,179)	143 (104,200)	121.5 (87,185.75)	< 0.001
CMV IgG: Positive	192 (99.48)	192 (99.48)	191 (98.96)	575 (99.31)	1
Framingham score—median(IQR)	0.02 (0.01,0.05)	0.05 (0.03,0.09)	0.12 (0.07,0.19)	0.06 (0.02,0.12)	< 0.001
CDK-Epi—median(IQR)	115.22 (99.67,130.88)	103.62 (92.52,119.16)	100.72 (84.9,113.08)	106.47 (92.65,120.7)	< 0.001
Baseline CD4 counts (cells/mL)—median(IQR)	533 (361.75,707)	536 (373,740)	560 (364.5,749.5)	538.5 (364,741.25)	0.8706
Nadir CD4 counts (cells/mL)—median(IQR)	215.5 (103.75,317.75)	238 (105,335)	177.5 (82,293.5)	213 (90,314)	0.054
Undetectable Viral Load: Yes	134 (68.72)	130 (66.33)	153 (78.46)	417 (71.16)	0.0196
Cumulative Viral Load—median(IQR)	12.22 (5.8,24.75)	17.88 (7.29,32.25)	17.97 (9.66,31.78)	15.34 (6.94,29.8)	0.002
cART use: Yes	171 (86.8)	176 (89.34)	178 (90.82)	525 (88.98)	0.4375
PI-containing regimen	94 (47.72)	102 (51.78)	114 (57.87)	310 (52.45)	0.1271
NNRTI-containing regimen	124 (62.94)	121 (61.42)	137 (69.54)	382 (64.64)	0.2006
Time on cART (years)—median (IQR)	2.82 (0.36,7.84)	3.98 (0.51,10.1)	5.18 (1.64,12.22)	4.18 (0.67,10.35)	< 0.001
Time on PI (years)—median (IQR)	0 (0,3.78)	0.1 (0,5.85)	1.99 (0,11.22)	0.15 (0,6.23)	< 0.001
Time on NNRTI (years)—median (IQR)	0.74 (0,3.68)	0.94 (0,3.38)	1.68 (0,4.41)	1.08 (0,3.81)	0.1025

^a^ Numbers are N (%) unless noted otherwise

Abbreviations: IQR—interquartile range; AMI—acute myocardial infarction; CVD—cardiovascular disease; BMI—body mass index; DM—diabetes mellitus; HDL—high density lipoprotein; LDL—low density lipoprotein; hs-CRP high sensitivity C-reactive protein; CMV—cytomegalovirus.

## Results

Of the 649 patients included in the study, 591 had a valid cIMT exam and were included in this analysis. The 58 patients without a valid exam were similar to the ones included in this analysis regarding all the variables analyzed (data not shown). As expected, the median of cIMT was significantly larger for men than women (0.56mm vs 0.53mm; p = 0.002; overall: 0.54mm). In order to compare cIMT with all variables considered in this study, we categorized cIMT values in terciles and the comparisons are shown in Table 1. Both traditional risk factors for CVD—such as all the components of the Framingham score and also HIV-specific factors, such as undetectable viral load, time on cART, time on IP-containing regimen, cumulative viral load and nadir CD4 counts (marginally) were associated with increased cIMT (Table 1).

In univariable linear regression analysis, sex, age, smoking, cumulative smoking, waist circumference, BMI, family history of sudden death, family history of AMI or stroke, history of CVD, use of lipid-lowering drugs, hypertension, DM, time on cART, time on IP-containing regimen, undetectable VL, total cholesterol, LDL, triglycerides, dyslipidemia and creatinine clearance were significantly associated with Box-Cox-transformed cIMT values. All variables in Table 1 except the Framingham score were considered for the multivariable model; after the best model was selected by the lasso procedure, sex, age, smoking, BMI, family history of AMI or stroke, and hypertension were still significantly associated, while LDL (p = 0.06) and use of lipid-lowering drugs (p = 0.07) were marginally associated, but still kept in the model. No first order interactions were significant among the variables in the final model. Table 2 presents the results for the proportional odds model with the same covariables selected in the linear models with the adaptive lasso procedure. Hypertension presented the strongest association with cIMT terciles (OR = 2.51; 95% CI = 1.69–3.73), followed by smoking (current; OR = 1,82; 95% CI = 1.19–2.79) and family history of AMI or stroke (OR = 1.60; 95% CI = 1.10–2.32).

**Table 2:** Predictors of cIMT determined from multivariable models selected after the adaptive lasso procedure.

Variable	OR (95% CI) ^a^	p-value ^a^	p-value—linear model ^b^
Sex: Female	0.56 (0.4–0.8)	0.001	<0.001
Age (per year)	1.12 (1.1–1.14)	<0.001	<0.001
Smoking	-	0.022	0.002
Former	1.16 (0.77–1.74)	-	-
Current	1.82 (1.19–2.79)	-	-
BMI	1.05 (1.01–1.09)	0.012	0.001
Family History of AMI or Stroke: Yes	1.6 (1.1–2.32)	0.013	0.001
Use of lipid-lowering drugs: Yes	0.73 (0.44–1.19)	0.205	0.056
Hypertension: Yes	2.51 (1.69–3.73)	<0.001	<0.001
LDL (mg/dL)	1 (1–1.01)	0.479	0.072

^a^ These results are from the proportional odds model

^b^ These results are from the linear model originally selected by the adaptive lasso procedure

Abbreviations: BMI—body mass index; LDL—low density lipoprotein.

Reference categories: Sex: Male; Smoking: Never; Family History of AMI or Stroke: No; Use of lipid-lowering drugs: No; Hypertension: No

## Discussion

We studied the association of several characteristics with carotid intima-media thickness in a large sample of HIV infected patients in Rio de Janeiro, Brazil. Our results show that traditional cardiovascular risk factors are the major players in determining increased cIMT in these patients, after controlling for other covariables. To our knowledge, this is one of the very few studies from low and middle income countries and one of the largest therein ever published on this subject.

Life expectancy of HIV-positive patients has dramatically improved with the advent of combination antiretroviral therapy [35]. As a consequence, a larger number of HIV infected individuals are living longer and facing the double challenge of HIV infection—with its lifelong treatment—and the increasing burden of chronic non-communicable diseases (NCDs) such as diabetes, cancer, cardiovascular diseases and renal failure [3–7].

Carotid IMT was found to be a strong predictor of future cardiovascular events, especially stroke and myocardial infarction [36], acting as a marker for subclinical atherosclerotic disease. In the search for an early atherosclerosis marker in HIV-infected patients, cIMT has been shown in several studies to reflect accurately atherosclerotic lesions and progression when compared with HIV negative controls [19,20,37].

The median thickness in our sample was somewhat lower than other reported studies. For a median age of 43.8 years the overall median was only 0.56mm, compared to values around 0.7mm among patients within similar age ranges for the common carotid measurements, the same region we studied [21,38,39]. Many studies use other regions, like the combination of common, bifurcation and internal carotid, which tend to yield higher values, especially if the maximum is employed [13,20,23]. These differences may be due to the lack of uniformity cIMT values are obtained, including specific location, measurement in both sides and use of median, average or maximum values within the region. For example, Fitch et al [38] report a mean of 0.68mm among HIV-infected patients with mean age of 44 years, but they used the measurement of the left common carotid only. Even though population characteristics also influence the measurements, we found it very hard to compare our raw measurements with the literature.

As expected, the cIMT median was significantly lower for women, and the variable sex was kept in all models. Since no interactions between sex and other variables kept in the final model were significant, we did not perform a separate analysis for women. Overall Framingham risk score was relatively low for our population (6%, Table 1), but presented a clear increasing gradient among the cIMT terciles. This may be because the score only takes into account traditional CVD risk factors, leaving other aspects such as immune activation, coagulation disorders, kidney disease, HIV itself and the role of cART out of the equation.

We studied the relationships of traditional and HIV-specific variables with cIMT. Even though both traditional and HIV-specific variables were associated with cIMT in a bivariate analysis (Table 1), after we adjusted the best model with the adaptive lasso procedure, only traditional variables were retained as risk factors (Table 2). These results are in agreement with recent studies that show the predominance of such factors in baseline analysis [23], including a study from Uganda [40]; they are also predominant when progression is taken into account [41,42], although some studies have been suggesting HIV-specific factors associated with cIMT progression [20,21].

Even though time on cART and time on PI-containing regimen were associated with cIMT, this association was confounded by the age of patients, which is associated both with time of ART use and the outcome. In fact in a model controlled by age alone, both variables became non-significant (data not shown).

Our results are also in agreement with another Brazilian study that compared several risk factors with subclinical atherosclerosis, where major factors were traditional ones; even though they also found HIV viral load as a risk factor, that was restricted to those older than 40 years of age (mean age of 47.8 years) and who were at low risk for other CVD [23]. We carefully assessed viral load as a cumulative variable, but after adjustment for traditional risk factors, it did not hold significance. This approach proved to be powerful for grab long run exposure to viral replication and which has been recently described as a risk factor for mortality [28].

We used the adaptive lasso procedure to select the best model because it has optimal properties for variable selection (oracle properties) and it has increasingly been used instead of more traditional techniques such as stepwise regression [33]. This approach allowed us to choose the final model that did not include the HIV-related variables and gives us confidence that this is the model that better explains the variation in cIMT among our patients.

In order to give a better grasp of the strength of association with covariables, we showed proportional odds models for the cIMT terciles where hypertension and current smoking had the strongest association. Considering a 10 year increase for age it yielded an OR of about 3.01, which can be considered as a major factor as well. This finding is also in agreement with a recent study that pointed age and smoking as a major factor for cIMT [38].

Our study has several limitations. First, the lack of an HIV negative control group did not allow us to study the association of HIV infection per se with cIMT and the fact that we are only analyzing baseline data did not allow us to study cIMT progression over time. One consequence is that we did not have information about years of evolution of DM, so the variable was defined for the moment of data collection only, even though we did took into account the value of glycated hemoglobin. The main strengths of our study include the large sample involved, the use of a pre-validated questionnaire and procedures from a large Brazilian study and the possibility of combining this with a large ongoing HIV clinical cohort in Rio de Janeiro.

## Conclusion

Our study showed that among HIV infected patients in Brazil traditional cardiovascular disease risk factors are the major players in determining increased cIMT. We believe our study is able to contribute important data to the knowledge of cardiovascular status among HIV infected patients in our country and highlights the critical need for establishing comprehensive HIV management programmes in low and middle income countries integrating the evaluation of cardiovascular diseases into HIV care, as has already been done in developed countries. Efforts to address smoking cessation, blood pressure control, and screening and treatment of lipid and glucose disorders need to remain at the forefront of our actions to ensure that the gains in the treatment of HIV infection translate into the most favorable long-term outcomes possible.
